# Cardiorespiratory fitness as a mediator between body fat rate and executive function in college students

**DOI:** 10.3389/fendo.2023.1293388

**Published:** 2023-12-18

**Authors:** Lina Zhu, Aihong He, Dandan Chen, Xiaoxiao Dong, Xuan Xiong, Aiguo Chen

**Affiliations:** ^1^ College of Physical Education, Yangzhou University, Yangzhou, Jiangsu, China; ^2^ College of Physical Education, Yangzhou Polytechnic College, Yangzhou, Jiangsu, China; ^3^ Nanjing Sport Institute, Nanjing, Jiangsu, China

**Keywords:** body fat rate, cardiorespiratory fitness, executive function, young adult, mediation effect

## Abstract

**Purpose:**

To examine whether body fat rate (BF%) is associated with cardiorespiratory fitness (CRF) and whether cardiorespiratory fitness (CRF) mediates the association between BF% and Executive function (EF) in young adults.

**Methods:**

In this cross-sectional study, 226 college students were recruited from an university. Flanker, 2-back, and odder and shifting tasks were used to assess EF. The incremental cardiopulmonary exercise tests were performed, and maximal oxygen consumption was recorded during test. The body composition measuring instrument was used to evaluate the participants’ BF%.

**Results:**

The BF% of college students was negatively correlated with each EF, BF% was negatively correlated with CRF, and CRF was negatively correlated with EF (*P*< 0.001). Structural equation modeling (SEM) and simultaneous analysis of several groups were used to construct mediator model. The CRF of college students plays a partial mediating role between BF% and EF, and the mediating effect accounts for 48.8% of the total effect value. Sex has no moderate effect on the relationship between BF%, CRF, and EF.

**Conclusions:**

College students with high BF% can improve their CRF by strengthening physical exercise, thereby indirectly improving their EF. Therefore, college students who have a higher body fat percentage should be compensated for engaging in physical exercise in order to enhance their CRF and mitigate the detrimental effects of obesity and overweight on EF.

## 1 Introduction

In recent years, significant changes have occurred in people’s lifestyles, including their dietary habits, work routines, and rest patterns, due to the improvement of the economic level (1). However, daily physical activities and exercise have gradually decreased, leading to an increase in the incidence of obesity, which has become a serious social and public health issue.

Executive Function (EF) refers to the advanced cognitive processes that individuals use to control and adjust other cognitive processes when completing complex cognitive tasks, including inhibition, updating, and shifting (2, 3). As the core of an individual’s cognitive, emotional, and intellectual activities, EF plays a crucial role in learning behavior, physical and mental health, and the development of future achievements (4, 5). Therefore, investigating the factors that affect the development of individual EF has become a forefront research topic.

High body fat is one of the important characteristics of obese and overweight individuals, and it is also a significant risk factor that affects the healthy development of individual EFs. Currently, the potential biological mechanism of the relationship between obesity and overweight and executive dysfunction is not clear. However, a large number of clinical and animal model studies suggest that impaired regulatory function of insulin (6), glucose (7), increased systemic and central inflammation (8, 9), brain structural damage (10), and abnormal functional network activity (11) may be the potential mechanisms for excessive body fat to cause individuals to perform abnormal functions. Additionally, studies have suggested that levels of fat-related sex hormones may moderate the performance of EFs (12–15). However, whether there are gender differences in the relationship between the two is still debated in the available evidence (15, 16), and further research is necessary. Studies have shown that high body fat in adulthood is associated with an increased risk of executive dysfunction and dementia in later age (17–19).

High body fat has a detrimental impact on cardiorespiratory fitness (CRF) in children and adolescents (20–22). CRF, also known as aerobic fitness and aerobic capacity, is a reflection of an individual’s physical health status and exercise ability. It encompasses the ability of the cardiovascular circulation system, respiratory system, and muscle system to absorb and utilize oxygen to perform work during the continuous increase in physical activity load. Studies have shown that obese individuals who engage in regular physical exercise exhibit better metabolic function than those who do not, indicating that maintaining higher CRF reduces the risk of obesity-induced metabolic and cardiovascular diseases (23, 24). Furthermore, CRF is closely linked to the healthy development of EFs. A growing body of evidence suggests that adolescents with higher CRF exhibit more effective abilities to suppress non-task-related interference information, better performance in working memory accuracy, and more sensitive task switching (25–30). Therefore, an individual’s CRF is affected by both body fat and EF, and body fat can directly affect EF performance. There is a pairwise correlation among the three. However, there is no study on the interaction among the three and whether there are sex differences in the relationship between the three. The 2014 report on the physical health of college students in China highlighted the rising rates of overweight and obesity among this population, as well as a decline in their physical fitness and cardiopulmonary function, resulting in various health, social, and psychological problems (31). Against this backdrop, this paper aims to explore the correlation between BF%, CRF, and EF of college students. Furthermore, a structural equation model is used to investigate the mediator effect of CRF between the percentage of body fat and EF, as well as gender differences. The findings of this paper will provide new evidence for fully understanding the relationship between obesity and EF of college students, as well as its potential influencing factors. Ultimately, this paper aims to lay a practical foundation for relevant departments to take preventive intervention measures to improve the physical and mental health of college students.

## 2 Methods

### 2.1 Participants

A total of 226 college students, ranging in age from 18 to 22, were recruited and chosen from an university located in Yangzhou. The sample consisted of 118 male and 108 female participants. The selection criteria included being right-handed as assessed by the Edinburgh Test (32) and Han nationality. Additionally, individuals with abnormal mental health levels as evaluated by Symptom Checklist-90-Revised (33), a history of mental disorders or genetic diseases, poor current mental condition, severe physical illnesses, brain trauma or nervous system diseases, as well as drug or alcohol dependence were excluded. The experiment was conducted in Yangzhou, China, with approval from the Ethical and Human Protection Committee of the Affiliated Hospital of Yangzhou University (2017-YKL045-01). Participants signed an informed consent form. All study procedures were in accordance with the latest version of the Declaration of Helsinki.

### 2.2 CRF test

The evaluation of individual CRF often relies on the measurement of maximum oxygen uptake (VO_2max_). In this particular study, a progressive load exercise program was implemented, wherein subjects underwent an incremental load exercise on a pedal power bicycle (Elmed EGT1000). The measurement of maximum oxygen uptake was conducted using a CORTEX METALYZER-II desktop gas metabolism analyzer, which is manufactured in Germany. The test results were then used to infer the individual’s CRF. Additionally, the change in heart rate during exercise was monitored using a POLAR heart rate band. Prior to commencing the experiment, all participants were mandated to complete the Physical Activity Preparation Questionnaire (PAR-Q) in order to ascertain their lack of prior exposure to vigorous exercise or consumption of substances that stimulate the nervous system within the 24 hours preceding the test. Additionally, all participants were engage in a preparatory activity lasting between 3 to 5 minutes to mitigate the risk of sports-related injuries. The baseline heart rate (HR) of each participant was recorded prior to the commencement of the test. The test officially commenced once the participants’ heart rates had returned to a state of rest. The initial power load of the power bicycle is set at 50 watts, and participants are required to maintain a rhythm of 55-60 revolutions per minute. Subsequently, the load is increased by 50 watts every 3 minutes until participants report exhaustion. VO_2 max_ is considered to be reached if any of the following criteria are met: (a) Oxygen intake remains relatively stable or slightly decreases (1500 ml/min) with the increase in load on the power bicycle. (b) Respiratory quotient is equal to or greater than 1.1. (c) Maximum heart rate exceeds 180 beats per minute. (d) Despite repeated encouragement, participants are unable to sustain a bicycle speed of 50-60 revolutions per minute. Following the completion of the test, the primary pathway documented the measurement, and the participants engaged in a period of rest lasting approximately 5 minutes. Subsequently, the subjects departed after confirming the absence of any anomalous reactions. The computation of CRF entailed the multiplication of the participants’ VO_2 max_ (L/min) by 1000, which was then divided by their weight (kg) to yield the relative maximum oxygen intake (ml/min/kg). This value serves as an indicator of the participants’ CRF.

### 2.3 BF% measurement

This study employed the Health Keeper-Xcan body composition measuring instrument, a human body composition measuring instrument produced by Bionet Company in South Korea, featuring 12 points of contact electrodes. The bioelectric impedance analysis and 6-frequency multiloop method were utilized to comprehensively assess various body components. Participants were instructed to hold a hand-held electrode and stand on the foot electrode for approximately 40 seconds to complete the test. Subsequently, immediate measurements of human body moisture, protein, muscle, and fat, as well as measurements of the left and right upper limbs and left and right lower limbs, were obtained. This study examines the criteria established by the World Health Organization (WHO) for evaluating obesity, as well as the commonly utilized criteria for determining overweight and obesity based on BF%.

### 2.4 Executive function test tasks

The computerized neuropsychological test battery, known as the assessment’s battery, comprises three tests designed to assess a range of abilities associated with EF. Previous studies have utilized the EF assessments battery, which can be administered in a group format. In the present study, we imposed a requirement for all participants to promptly press the button, while simultaneously ensuring accuracy. The determination of outcome parameters was based on reaction time (RT).

Inhibition performance was evaluated using a modified Erickson Flanker task, a task known for its sensitivity to the effects of both acute and chronic exercises (34–36). The participants were provided with instructions to respond promptly and accurately to each trial by selecting the center letter in each array. Any incorrect responses, including pressing the wrong button or responding within 150 ms or timeout, were deemed as errors. Prior to the main task, participants were required to complete 12 practice trials. The main task consisted of two blocks, each comprising 48 trials, with a one-minute rest period between the blocks. The congruent and incongruent trials were presented in a random order, with an equal likelihood of occurrence in each block. The overall duration of the task was approximately six minutes. The calculation of the difference in response times involved subtracting the reaction times for the congruent trail from those of the incongruent trail. A smaller difference in reaction time and a higher accuracy rate indicated superior inhibition performance.

In a previous study conducted by our researcher group (37), the 2-back task was employed. The 2-back condition served as a working memory task. If a participant’s task performance fell below 60% accuracy (ACC), the same practice was repeated. The mean reaction time (RT) was used to evaluate the behavioral performance in this working memory (WM) task, with shorter RT indicating better WM performance.

More-odd shifting task was used to assessment the shifting. Participants were asked to switch back and forth between two different tasks that used the same numeric stimuli, which appeared in the center of the computer screen (38). Participants received three blocks of stimuli. The first two blocks were the homogenous conditions in which only one task was performed. The task contained two conditions. In one condition, participants determined whether the digit presented was greater or less than 5. In the other condition, participants determined whether the digit presented was odd or even. During each trial, a solid or dashed outline cue appeared simultaneously with the numeric stimuli instructing participants to make a decision (greater/less than 5, or odd/even). The two blocks were counterbalanced across participants. The third block consisted of the task-heterogeneous condition in which participants were required to switch between equiprobable task sets on some trials and repeatedly perform the same task over trials in other cases. The two tasks alternated randomly in the heterogeneous block, with a maximum of seven consecutive trials performed repeatedly for each task. Therefore, all trials in the heterogeneous block were categorized into either switch or non-switch conditions. White numeric stimuli were presented on a black background for 200 ms, with a 2000 ms interstimulus interval from stimulus offset to onset. Participants completed 50 and 256 trials in homogenous and heterogeneous conditions, respectively.

### 2.5 Statistical analysis

The data obtained in this paper were analyzed using SPSS25.0 statistical software, which facilitated the execution of independent sample t-tests and Pearson correlation analysis. The validation of the structural equation model was conducted using Amos 25.0, and further analysis was performed on the multi-group structural equation model. Additionally, the significance of the intermediate effect was assessed using the Bootstrap method, with a statistical significance level set at *P<* 0.05.

## 3 Results

### 3.1 Behavioral performance and correlated analysis

According to obesity and overweight criteria (39, 40), men are considered overweight if their BF% falls within the range of 20% to 25%, while a BF% exceeding 25% indicates obesity. For women, a BF% between 25% and 30% is classified as overweight, while a percentage exceeding 30% is indicative of obesity. The study findings reveal that out of the total sample, 37 men were classified as overweight, while 20 men were categorized as obese. Similarly, 36 women were identified as overweight, whereas 22 women were classified as obese.

Sex differences in BF%, CRF, and EF sub-functions RT of college students were analyzed by using an independent sample t-test. It was found that there were sex differences in VO_2 max_ (P< 0.001), BF% (*P<* 0.001), CRF (*P<* 0.001), and updating RT (*P* = 0.017), BMI (*P* = 0.192), inhibition RT (*P* = 0.055), and Shifting RT (*P* = 0.088) (see **Table 1**).

**Table 1:** Behavioral performances of BF%, CRF and EF sub-functions in college students.

	Males(N=118)		Females(N=108)		Total(N=226)		t
	M	SD	M	SD	M	SD	
Age (years)	18.92	0.98	19.25	0.89	19.08	0.95	-2.612
BMI(kg/m^2^)	21.67	3.13	21.11	3.23	21.41	3.18	-1.31
VO_2max_(L/min)	1.53	0.43	1.01	0.33	1.28	0.47	-10.04^***^
BF%(%)	19.96	5.26	25.05	5.65	22.39	6.00	7.02^***^
CRF(ml/min/kg)	23.16	6.32	18.44	6.26	20.90	6.71	-5.63^***^
Inhibition(ms)	13.96	14.22	17.66	14.59	15.73	14.48	1.93
Updating(ms)	1098.84	205.38	1169.67	237.29	1132.69	223.54	2.40^*^
Shifting(ms)	324.78	104.98	348.32	101.06	336.03	103.57	1.71

* means P < 0.05 and *** means P < 0.001. BMI, Body mass index, **BF%,** Body fat percentage; CRF, Cardiorespiratory fitness; M, Mean; SD, Standard deviation.

**Figure 1:**
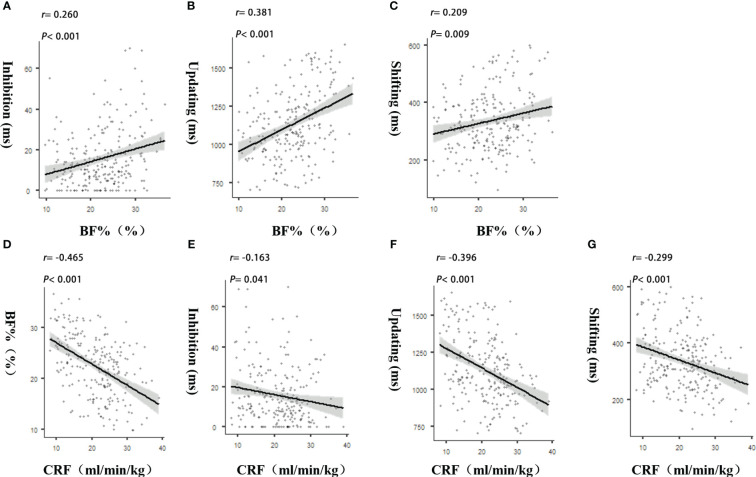
**(A–C)** showed the correlations among BF% and EF sub- functions; **(D)** showed the correlation between CRF and BF%; **(E–G)** showed the correlations among CRF and EF sub- functions.

Pearson correlation analysis showed that the BF% was positively correlated with the RT of each sub-function of EF; BF% is negatively correlated with CRF. CRF is negatively correlated with the RT of each sub-function of EF, and the correlation is statistically significant (see **Table 2** and **Figure 1**).

**Table 2:** Correlation coefficients of BF%, CRF and EF sub functions.

	BF%	CRF(ml/min/kg)	Inhibition(ms)	Updating(ms)	Shifting (ms)
BF%	1				
CRF(ml/min/kg)	-0.465^***^	1			
Inhibition(ms)	0.260^***^	-0.163^**^	1		
Updating(ms)	0.381^***^	-0.396^***^	0.206^**^	1	
Shifting(ms)	0.209^**^	-0.299^***^	0.185^**^	0.287^***^	1

The data in the table are correlation factors. ** means P < 0.01, *** means P < 0.001. BF%, Body fat percentage; CRF, Cardiorespiratory fitness.

### 3.2 Mediating effect of CRF between BF% and EF

**Figure 2:**
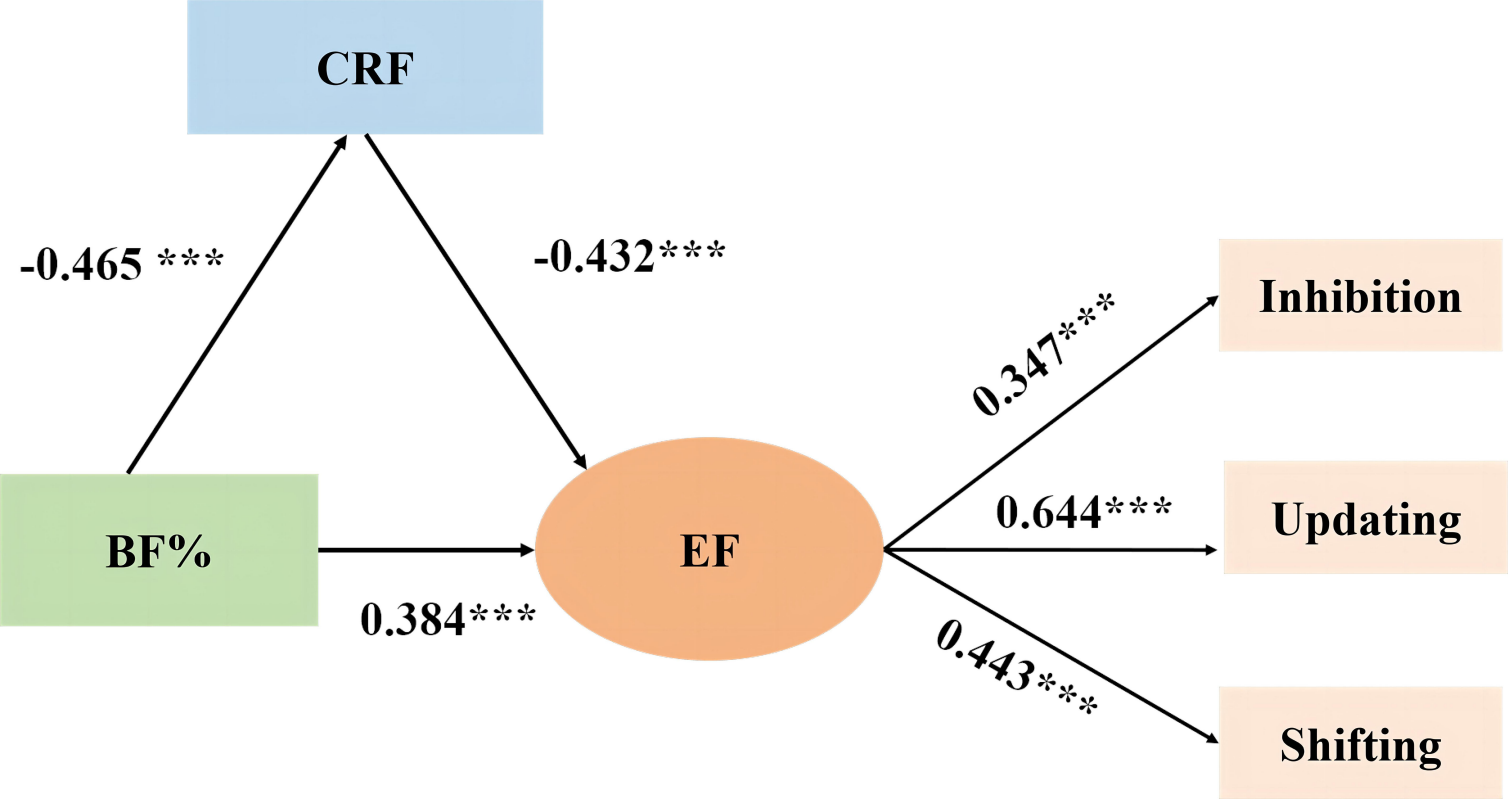
The Mediation Effect of CRF between BF% and EF in College Students.*** means P < 0.001. The numerical values in the figure represent normalized path coefficients (direct effects).

Structural equation modeling was used to establish a mediating model to test the role of mediating variables. The model included one latent variable, EF (taking inhibition, updating, and shifting as observation variables), and two explicit variables, BF% and CRF. The mediating effect was tested by the Bootstrap method of deviation correction. The fitting index of the model is χ2/df=1.252, GFI = 0. 992, NFI = 0. 961, CFI = 0. 993, RMSE = 0. 033, which indicates that the model fits well. BF% and CRF accounted for 48.8% of the variation in EF (*R^2 ^= *0.488). The mediation model path is shown in **Figure 2**. The total effect of BF% on EF was 0.584 [*P<* 0.001, 95% confidence interval (*CI*): 0.420 ~ 0.757], and the direct effect was 0. 384 (*P*<0.001, *95% CI*: 0.195 ~ 0.583), and the mediating effect was 0.201 (*P<* 0.001, 95% *CI:* 0.120 ~ 0.302), accounting for 34.41% of the total effect. Therefore, the BF% not only directly affects the EF of college students but also indirectly affects it by reducing their CRF; that is, CRF plays a partial mediating role between the BF% and EF.

### 3.3 Moderating effect of sex in the relationship among BF%, CRF and EF

Multi-group analysis was used to compare the relationship between EF, CRF, and BF% among different sex college students. When all the path coefficients were equal for different sex, the model fitted well. Compared with the unrestricted model, Δχ2/df (12.985) and Δdf (11) had no statistical significance (*P*= 0.294>0.05). The two models can be regarded as equal models (see **Table 3**). The mediating model has identity among different sex, which indicates that sex has no moderating effect on the relationship between BF%, CRF, and EF in college students.

**Table 3:** Multiple group structural equation model fitting indicators.

Models	χ^2^	df	*P*	χ^2^/df	GFI	NFI	CFI	RMSEA
Unrestricted	7.024	8	0.534	0.878	0.988	0.949	0.999	0.000
Restricted	20.008	19	0.394	1.053	0.966	0.854	0.991	0.015

χ^2^, chi-square value; df, degrees of freedom; χ2**/**df, ratio of the chi-square value to the degree of freedom; GFI, Goodness of fit index; NFI, Normed fit index; CFI, comparative fit index; RMSEA, Root mean square error of approximation.

## 4 Discussion

### 4.1 Correlations among BF%, CRF and EF in college students

This study revealed a significant disparity in the BF% between males and females, with males exhibiting lower levels. Conversely, no notable discrepancy in BMI by sex was observed, indicating incongruity between BMI and BF% as measures of obesity. This finding aligns with previous research, which similarly demonstrated limited agreement between BMI and BF% in assessing obesity among college students. Consequently, it is recommended that a comprehensive approach be adopted in practical settings to effectively identify and prevent obesity among college students. Nonetheless, the author acknowledges a limitation in the study. However, the author also highlights that the assessment of obesity relies on the measurement of BF%. By excluding the weight of muscle and other tissues, this measurement effectively discerns whether the increase in body mass is attributable to fat or muscle and other tissues, thereby providing a more precise reflection of the overall degree of obesity in individuals (39). Furthermore, this study additionally establishes the existence of sex disparities in CRF, with males exhibiting higher levels compared to females. The findings of this study confirm previous research indicating that men possess inherent advantages over women in terms of the oxygen transport system and muscle tissue content, resulting in superior performance in CRF (41). Furthermore, within the scope of EF analysis in college students, it is noteworthy that only the updating sub-function displays notable sex disparities, wherein males exhibit superior overall performance in comparison to females. Prior research has indicated that females tend to exhibit advantages in inhibition and shifting prior to reaching puberty, yet this sex disparity gradually diminishes after the onset of puberty (42). Voyer et al. discovered that the impact of sex differences on visuospatial working memory is contingent upon age and the specific evaluation tasks employed (43). Nevertheless, men consistently exhibit superior performance in visuospatial working memory. While certain studies have failed to identify any significant sex disparities in updating abilities, males consistently outperform females in this domain (15). Consequently, it is evident that sex exerts distinct effects on the inhibition, updating, and shifting aspects of EF among college students.

### 4.2 The mediating role of CRF in the relationship between BF% and EF

The present study revealed a negative association between the BF% and CRF, as well as a positive association between the BF% and sub-functional RT performances of EF. Additionally, CRF was found to negatively predict sub-functional RT performances of EF. This finding aligns with Rauner et al.’s (2013) systematic review, which highlighted the inverse relationship between fat content resulting from overweight or obesity and CRF (44). Previous research has established that overweight or obesity significantly impacts both CRF and EF (45, 46), a finding that aligns with our own study results. Hence, our findings corroborate the established association between BF%, CRF, and EF as observed in prior research. Specifically, our results indicate that higher BF% in college students are linked to poorer CRF levels. Moreover, elevated BF% are associated with diminished performance across various sub-functions of EF. Conversely, higher levels of CRF are associated with improved performance in each sub-function of EF. The relationship between CRF and the performance of individual sub-functions of EF is positively correlated, with higher levels of CRF associated with improved performance. While previous research has examined the association between BF%, CRF, and EF, this study is the first to investigate the relationship between the three sub-functions of EF, BF%, and CRF specifically in college students.

This study employed structural equation modeling to establish a well-fitting mediating effect model, examining the relationship between BF%, CRF, and EF. The findings revealed that CRF partially mediated the association between BF% and EF in college students. This suggests that the impact of body fat on EF is not only direct but also indirect, mediated by CRF. Furthermore, the present study discovered that sex did not exert any influence on the association between BF%, CRF, and EF, but failure to detect a difference does not establish the absence of a sex difference. It is worth noting that elevated BF% are considered significant indicators of both obesity and overweight, and the detrimental impact of obesity on cognitive function has been substantiated across various age groups (47–49). Meanwhile, an inverse relationship between EFs and % body fat cannot be disregarded. Obese individuals exhibit traits such as a strong inclination toward immediate gratification in their pursuit of appetizing food, as well as a limited ability to regulate this motivational inclination, resulting in challenges when confronted with external stimuli promoting palatable food (50). Recently observation has also posited that a higher baseline EF capacity may be linked to a more advantageous trajectory in terms of BMI and waist circumference. Conversely, a low EF at follow-up is correlated with obesity and increased uncontrolled eating (51). Consequently, identifying individuals with low EF could prove beneficial in identifying adolescents who are at risk of obesity at earlier stages. This supplementary information substantiates the importance of considering cognitive training strategies when making decisions regarding interventions for weight loss.

Currently, numerous studies have corroborated the beneficial influence of CRF on EF. For instance, Themanson (2006) discovered that young adults in the high fitness group exhibited superior monitoring skills for irrelevant and erroneous information compared to their low fitness counterparts, indicating enhanced inhibition abilities (52). According to Scott (2016), there exists a positive correlation between CRF levels and the efficacy of neural functioning within the frontal-parietal lobe network, ultimately leading to improved updating performance (28). Additionally, both Scisco (2008) and Themanson (2008) discovered that young adults belonging to the high fitness group exhibited superior shifting abilities compared to their counterparts in the low fitness group (27, 53). Neuroimaging studies have indicated that individuals who are overweight or obese exhibit notable alterations in gray matter structure and white matter fiber density, resulting in diminished cognitive performance compared to those with normal body fat levels (11, 54, 55). CRF serves as a mediating variable influencing the relationship between obesity and academic achievement (53, 54). In recent academic discourse, the notion of “obesity but health” has been introduced by researchers. Notably, the Sardinha study revealed that adolescents categorized as having high CRF but normal weight, as well as those with high CRF but overweight or obesity, exhibit superior academic performance when compared to their counterparts with low CRF (56, 57). Consequently, this study posits that the substantial proportion of body fat could potentially exert a detrimental influence on the EF of children and adolescents; however, the presence of enhanced CRF may mitigate or diminish this adverse effect.

There are limitations on interpreting the causality of the mediated moderation effect model because our study used the cross-sectional study design. Although previous evidence supports a rapid decline tendency of EF in obesity, we cannot conclude any causal effects of an inactive lifestyle on the neuropsychological processes observed in the current study. These findings can potentially serve as an initial step for future longitudinal and interventional investigations, aiming to provide additional insights into the temporal association between obesity and intricate cognitive processing in the developing brain, as well as to establish a cause-and-effect relationship. Another important consideration is that there may be additional factors, not examined in this study, that could potentially contribute to the observed relationship. Although we excluded participants with physical activity contraindications, these obesity individuals allocate a greater amount of time to the utilization of electronic devices and engagement in professional courses, it is possible that at least some of the included participants had ongoing pathological processes that are still not symptomatically expressed and therefore they were not aware of.

## 5 Conclusion

CRF plays a crucial role in mediating the relationship between BF% and EF. It is suggested that CRF may determine the adverse impact of BF% on EF. Therefore, for college students with high body fat or obesity, it is imperative for colleges and universities to focus on reforming the physical education curriculum while also emphasizing the training and promotion of CRF in the curriculum design. This approach can effectively mitigate the negative impact of overweight and obesity on college students’ EF.

## Data availability statement

The original contributions presented in the study are included in the article/supplementary material. Further inquiries can be directed to the corresponding author.

## Ethics statement

The studies involving humans were approved by Affiliated Hospital of Yangzhou University. The studies were conducted in accordance with the local legislation and institutional requirements. The participants provided their written informed consent to participate in this study.

## Author contributions

LZ: Conceptualization, Data curation, Formal Analysis, Funding acquisition, Investigation, Methodology, Writing – original draft, Writing – review & editing. AH: Formal Analysis, Investigation, Methodology, Project administration, Visualization, Writing – original draft, Writing – review & editing. DC: Investigation, Methodology, Validation, Visualization, Writing – review & editing. XD: Formal Analysis, Investigation, Methodology, Validation, Writing – review & editing. XX: Formal Analysis, Investigation, Methodology, Writing – review & editing. AC: Conceptualization, Funding acquisition, Project administration, Resources, Supervision, Writing – review & editing.
